# Supplementary material for: Single-molecule sequencing detection of N6-methyladenine in microbial reference materials
Source: Nat Commun. 2019 Feb 4;10:579. doi: 10.1038/s41467-019-08289-9 (PMC6362088; doi:10.1038/s41467-019-08289-9)
Supplement: Supplementary file 1 — Supplementary Information [file 41467_2019_8289_MOESM1_ESM.pdf]

Supplementary Information

Single-molecule sequencing detection of *N*6-methyladenine in microbial reference materials

McIntyre et al.

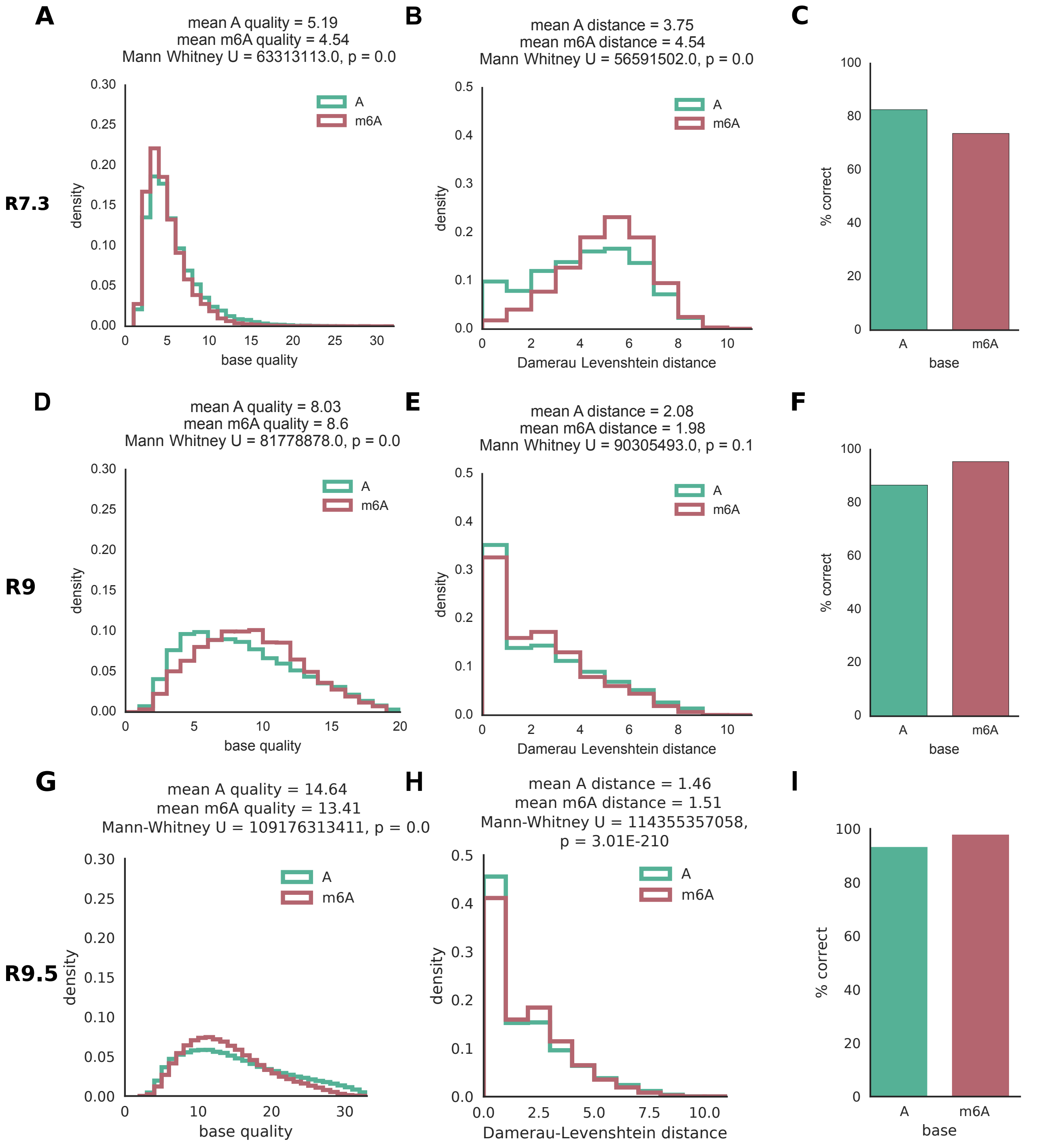

Supplementary Figure 1. Methylation and base quality. The effects of adenine methylation on read quality and alignment vary across flowcell versions (**A-C**: R7.3, **D-F**: R9, **G-I**: R9.5). Base quality **(A,D,G)** and Damerau-Levenshtein distance **(B,E,H)** between the surrounding 11-mer and an aligned reference showed little change with methylation. With later versions, a slightly higher percentage of modified adenines are correctly basecalled **(C,F,I)**.

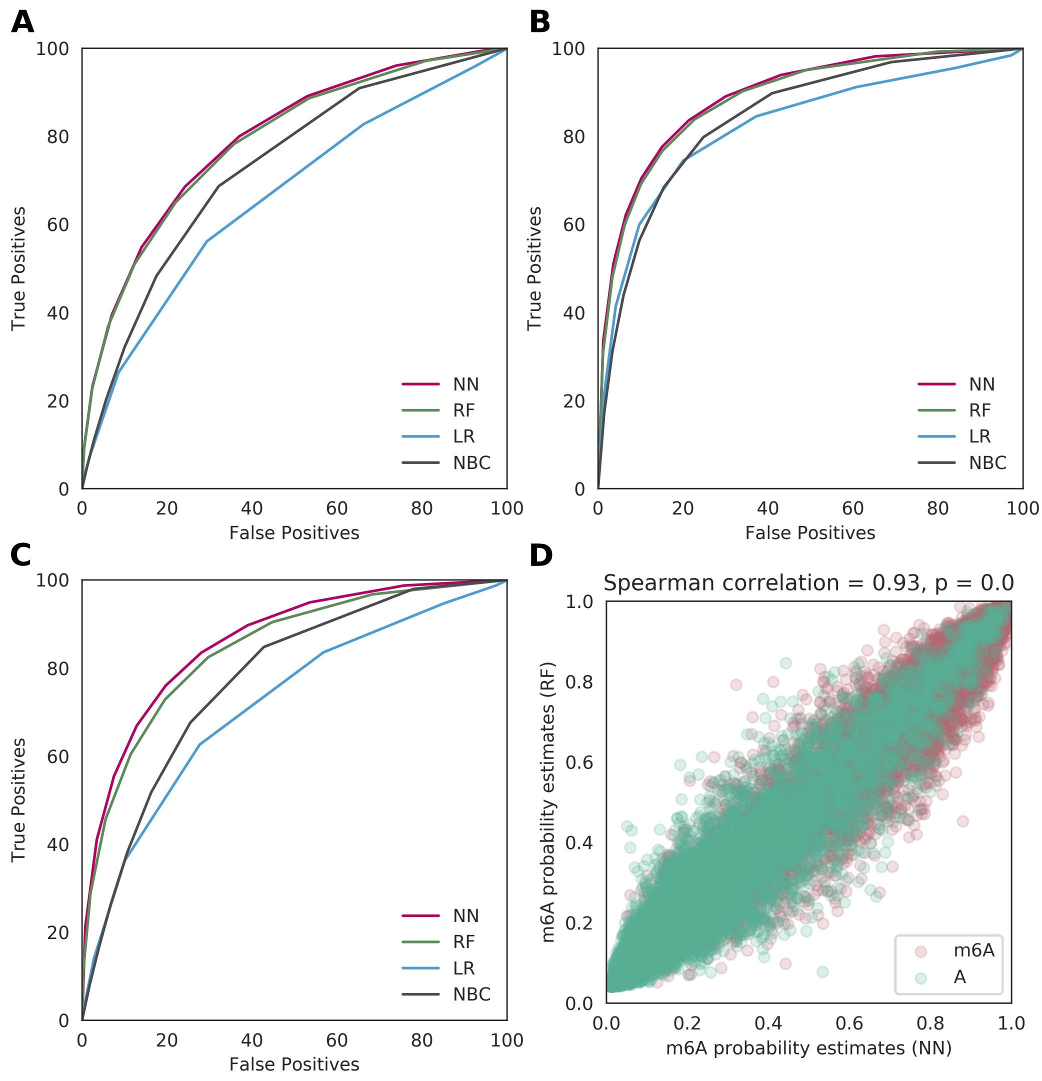

Supplementary Figure 2. Model performance with different numbers of features. ROC plots for a support multi-layer perceptron neural network (NN), random forest classifier (RF), linear regression classifier (LR), and naïve Bayes classifier (NBC) using **(A)** 7-mer, **(B)** 11-mer, and **(C)** 15-mer contexts to classify. **(D)** A comparison of the probability scores for 10,000 randomly selected classifications per base using the top (x-axis, neural network) and second place (x-axis, random forest classifier) algorithms for 11-mer contexts. Models were generated using one R9 experiment and tested on data from a second.

# Features	Classifier	Accuracy	AUC
4	NN	72.2	0.798
4	RF	71.5	0.792
4	LR	63.4	0.671
4	NBC	65.4	0.739
6	NN	81.3	0.895
6	RF	80.8	0.890
6	LR	77.2	0.828
6	NBC	76.5	0.848
8	NN	78.2	0.865
8	RF	76.7	0.848
8	LR	67.5	0.726
8	NBC	67.7	0.782
6	NN_sk	77.8	0.862
6	NN_hq_sk	81.8	0.897
6	NN_hq	84.2	0.920
6	NN_pos	95.4	0.992

Supplementary Table 1. Model performance with different features and read qualities. Accuracy and area under the curve (AUC) for methods and parameters tested (number of features and filters) in Figure 2C and Supplementary Figure 2. Methods include neural network (NN), random forest (RF), linear regression (LR), and naïve Bayes classifer (NBC), and filters allowing up to two skips (sk), only high-quality reads (hq) of QV>9, and positions (pos) covered by 15 or more reads.

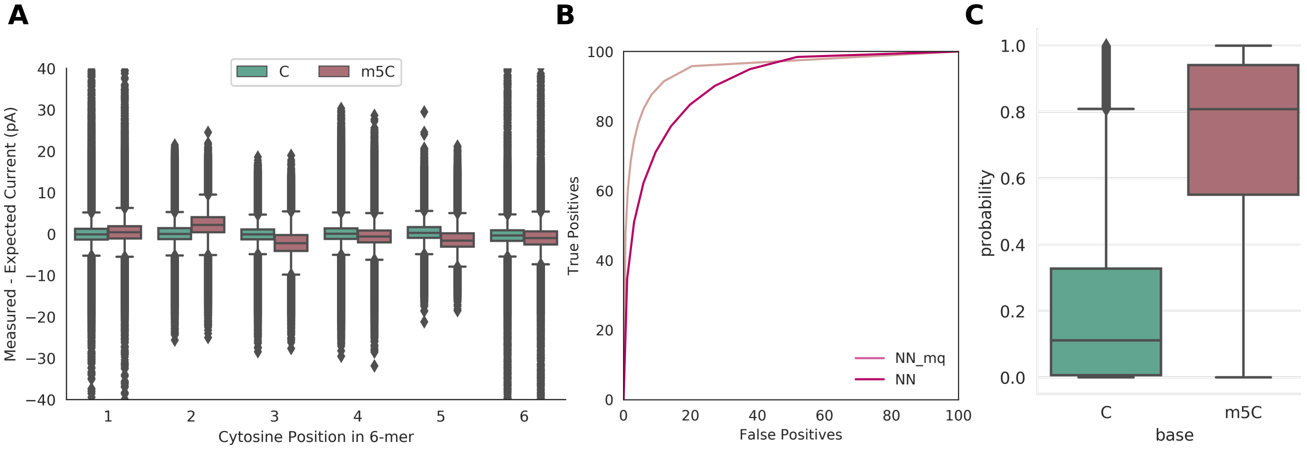

Supplementary Figure 3. Cytosine methylation detection. (A) Deviations between measured and expected current values across different positions in a 6-mer, depending on cytosine methylation status (outliers are truncated at +/- 40 pA). The variances for both methylated and unmethylated cytosines are higher than for m6A, likely in part because the reads were of lower average quality (5.64 for the M.SssI dataset and 6.41 for the PCR dataset, vs. 7.65 for the Chiu dataset and 7.32 for the Mason dataset). **(B)** A ROC plot shows m5C classifications with per-read accuracy of 82.2%, increasing to 87.1% for "medium quality" data ("mq", > mean base quality score of 7), similar to m6A despite including more sites with multiple methylated bases. **(C)** Probability scores for the detection of m5C using the same neural network classifier trained and tested using any quality reads from M. SssI-modified data and whole genome amplified data. Boxplot centre lines show medians and whiskers 1.5x interquartile range.

**Species** (# putative m6A MTase genes)	**Motif**	**Mean IPD Ratio**	**Total sites in genome**	**Detected in 2nd PacBio run?**	**PacBio + MeDIP-seq**	**PacBio + ONT**	**Sites detected by PB + ONT + MeDIP-seq**	**Sites missed by all methods**
B. subtilis (2)	CNC**A**NNNNNNNRTGT	5.96	535	yes	516	291	288	3
	AC**A**YNNNNNNNTGNG	5.76	535	yes	516	496	482	3
	none	2.01	6296		547	974	93	
E. coli (4)	AAG**A**NNNNNCTC	5.39	337	no data	153	191	97	0
	G**A**GNNNNNTCTT	6.03	337	no data	155	270	129	0
	G**A**TC	5.67	39872	no data	24529	36805	12181	14
	none	2.17	5054		1713	791	165	
E. faecalis (4)	CA**A**YNNNNNNTTYG	5.40	861	yes	830	857	827	0
	CRA**A**NNNNNNRTTG	5.17	861	yes	830	517	505	1
	CTKV**A**G	2.55	5909	CTBV**A**G	1788	1167	576	1246
	CTCC**A**G	2.80	478		205	89	54	48
	none	1.84	7638		1540	1493	334	
L. monocytogenes (2)	GC**A**NNNNNNNTGC	5.92	906	yes, also GC**A**AVDNNNNTGC	886	570	562	0
	ANAR**A**GTANYR	0.75	485	no	4	0	0	377
	none	2.00	7438		626	1264	105	
P. aeruginosa (1)	none	2.47	939		67	174	15	
S. aureus (4)	TCT**A**NNNNNNTTAA	6.74	425	yes	408	267	259	3
	TT**A**ANNNNNNTAGA	6.37	425	yes	408	3	3	5
	GA**A**GNNNNNNTTRG	6.57	230	yes	197	104	90	1
	CYA**A**NNNNNNCTTC	5.63	230	yes	199	156	144	2
	G**A**TCGVNY	2.23	399	G**A**TCBVNYD	17	195	17	11
	none	2.03	4259		423	812	87	
S. cerevisiae	none	2.64	1714		233	183	28	
S. enterica (3)	G**A**TC	5.85	37910	yes	24007	33632	11885	172
	CAG**A**G	6.43	5819	yes	2048	3053	1111	110
	BATGC**A**TV	3.81	730	BATGC**A**T	169	239	110	248
	none	2.47	2215		852	290	88	

Supplementary Table 2. Predicted m6A motifs and non-motif sites predicted by PacBio across eight species. A total of fourteen motifs were predicted as methylated (nineteen including methylated reverse complements). IPD = inter-pulse duration, a measure of the rate of nucleotide addition during sequencing. mCaller was used for ONT predictions.

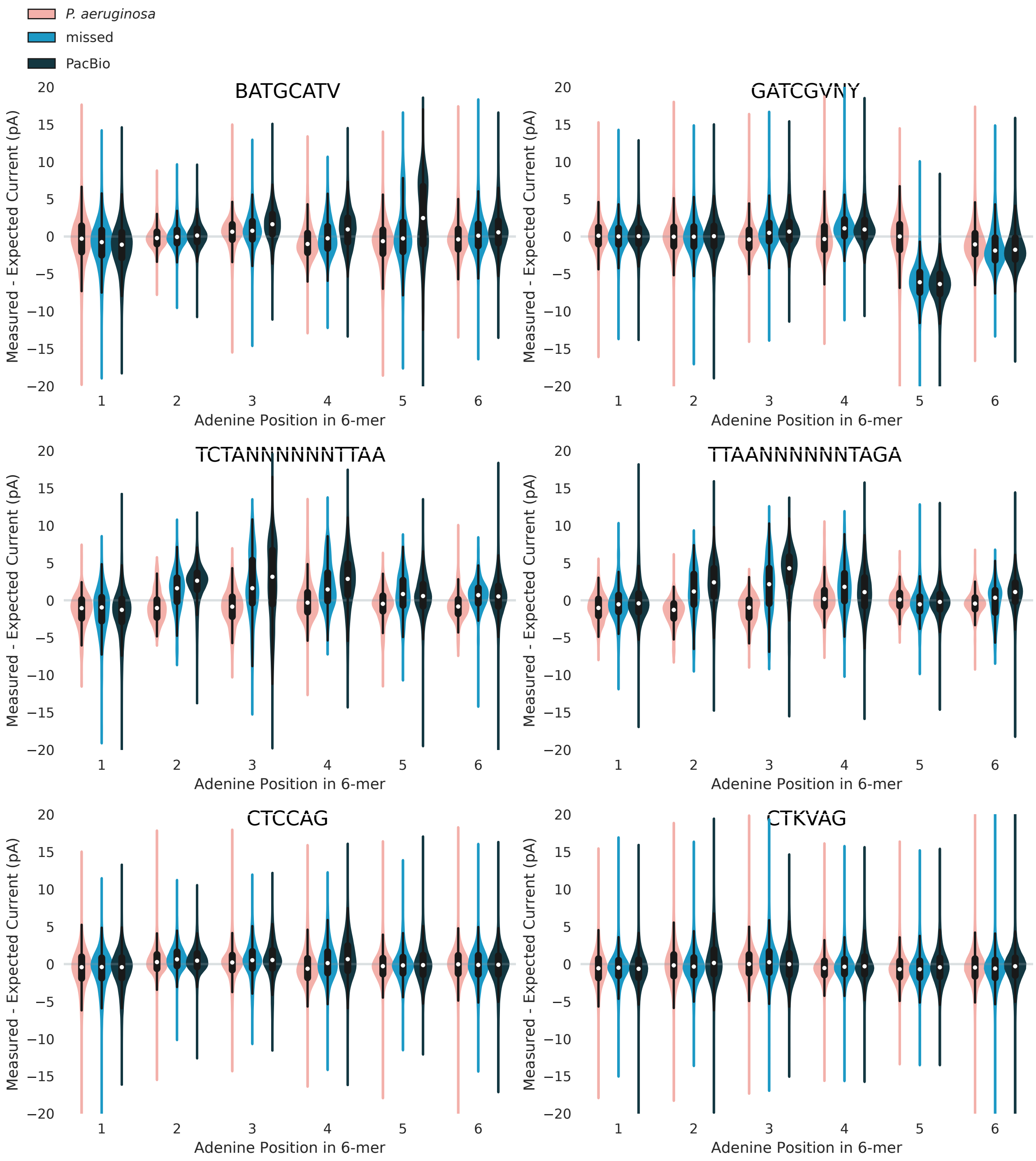

Supplementary Figure 4. Current differences among motifs. Plots of current differences from model values at motifs sites identified as methylated using PacBio sequencing, motif sites missed by PacBio in the same strain, and unmethylated sites for the same motif in *P. aeruginosa.* Inner boxplot centre lines show medians and whiskers 1.5x interquartile range.

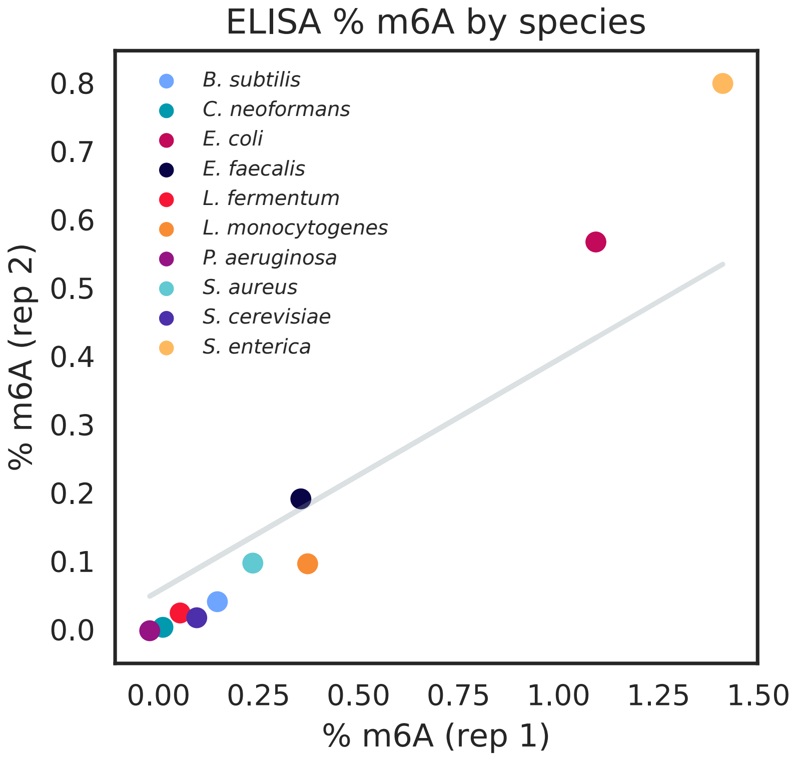

Supplementary Figure 5. Comparison of ELISA replicates. The percent m^6A in ten reference community strains (including two for which we did not have PacBio data – *C. neoformans* and *L. fermentum*), as measured using two replicates of a commercial ELISA kit. Pearson R = 0.60, p = 0.067; Spearman ρ = 0.16, p = 0.65.

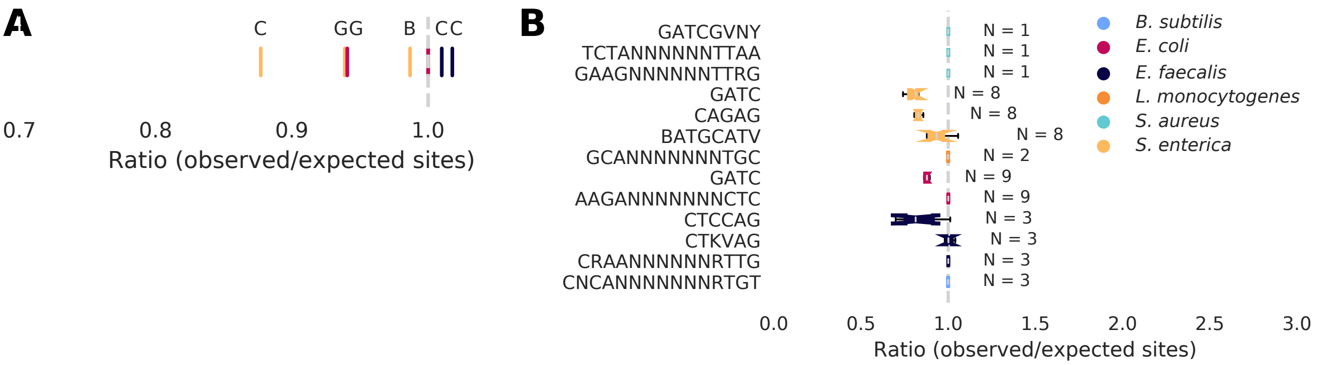

Supplementary Figure 6. Motif depletion calculated with Kr. Ratio of observed over expected motif counts calculated by Kr in **(A)** assembled genomes, colored by species and labeled by first nucleotide where there were multiple motifs, and **(B)** in predicted prophages within assembled genomes. Boxplots centre lines show medians, notches confidence intervals, and whiskers 1.5x interquartile range.

Kr, expressed in counts instead of frequencies:

$$Kr(m) = L(g)^{-1^{L(m)}} \prod_{s \in S} N(s)^{-1^{(L(m)-L(s))}}$$

where
m = motif of interest
S = the set of all submotifs of m (including m)
$N(s)$ = count of submotif s in the genome or sequence of interest
g = genome or sequence of interest
$L(x)$ = length of x

Species	PacBio		Illumina (WGS)		Illumina (MeDIP-seq)		Illumina (WGBS)	ONT	
	FDA Site (RSII)	UF (Sequel)	FDA Site (HiSeq4000)	WCM (iSeq)	WCM (iSeq)	WCM (NextSeq)	WCM (HiSeq)	UCSF	WCM
B. subtilis	X	X	X		X		X		X
E. faecalis	X	X	X			X	X		X
E. coli	X		X			X	X		X (x2)
L. fermentum				X		X	X		X
L. monocytogenes	X	X	X			X	X		X
P. aeruginosa	X		X			X	X		X
S. enterica	X	X	X			X	X		X
S. aureus	X	X	X			X	X		X
C. neoformans				X (x2)		X	X		X
S. cerevisiae	X	X		X (x2)		X	X		X
**NASA *E. coli* (K12)**						X	X	X	X
**Pool**				X					

Supplementary Table 3. Summary of data generated for the samples discussed in the manuscript. The NASA *E. coli* K12 ONT sequencing included lambda phage and mouse DNA.

Assembly, contig	Identified match	Pearson's r
B. subtilis, contig 1	B. subtilis subsp. spizizenii W23	0.99866
B. subtilis, contig 2	B. subtilis subsp. spizizenii TU-B-10	0.89454
C. neoformans	C. neoformans var. neoformans B-3501A	0.97109
E. coli, contig 1	E. coli str. K-12 substr. MG1655	0.99977
E. coli, contig 2	E. coli O104:H4 str. 2009EL-2050 plasmid p09EL50	0.97142
E. faecalis	E. faecalis OG1RF	0.99957
L. fermentum	L. fermentum F-6	0.99843
L. monocytogenes	L. monocytogenes SLCC2378	0.99990
P. aeruginosa	P. aeruginosa SCV20265	0.99990
S. aureus, contig 1	S. aureus subsp. aureus ECT-R 2	0.99948
S. aureus, contig 2	S. aureus subsp. aureus TW20 plasmid pTW20_1	0.60116
S. cerevisiae	S. cerevisiae BY4742	0.99955
S. enterica, contig 1	S. enterica subsp. enterica serovar Choleraesuis str. SC-B67	0.99974
S. enterica, contig 2	S. enterica subsp. enterica serovar Choleraesuis str. SC-B67 plasmid pSCV50	0.99122

Supplementary Table 4. Similarity to published genomes. Closely related genomes and plasmids inferred with ANI value Pearson's correlations. Note the lower Pearson's r for the *S. aureus* plasmid, which implies it is significantly different from previously known plasmids in this species.

Supplementary note: the *L. fermentum* genome was assembled using Illumina iSeq100 and nanopore data with a custom pipeline. Error corrected Nanopore and HiSeq reads were assembled using SPAdes[1]. Scaffolding of the assembled contigs was done using SSpace[2]. and gap filling was executed using GapFiller[3].

	Assembly statistics			Related strain statistics		
**Species**	**BUSCOs, %**	**GC, %**	**Length, bp**	**BUSCOs, %**	**GC, %**	**Length, bp**
B. subtilis	100.0	44.0	4 090 859	100.0	43.9	4 027 676
C. neoformans	62.1 (58.3)	48.3	27 181 434	96.9	45.6	19 699 782
E. coli	98.0 (93.9)	50.5	4 895 096	98.7 (98.0)	50.7	4 750 926
E. faecalis	97.3 (95.9)	37.6	2 865 554	97.3	37.8	2 739 625
L. fermentum	99.3	52.3	1 905 487	98.0 (97.3)	51.7	2 064 620
L. monocytogenes	100.0 (99.3)	38.0	3 012 754	100.0	38.0	2 941 360
P. aeruginosa	100.0	66.2	6 811 589	100.0	66.3	6 725 183
S. aureus	98.6	32.8	2 785 696	98.6	32.9	2 759 125
S. cerevisiae	96.2 (87.6)	38.1	12 843 354	99.0 (92.1)	38.1	12 147 068
S. enterica	98.6	52.2	4 852 168	98.7 (98.0)	52.2	4 805 258

Supplementary Table 5. Benchmarking Universal Single-Copy Orthologs. Percentages of BUSCOs identified in the genomes, GC content and total assembly lengths . The primary value is the percentage of all found BUSCOs, the value in the parentheses is the percentage of contiguous single-copy BUSCOs (where absent, is equal to the primary value).

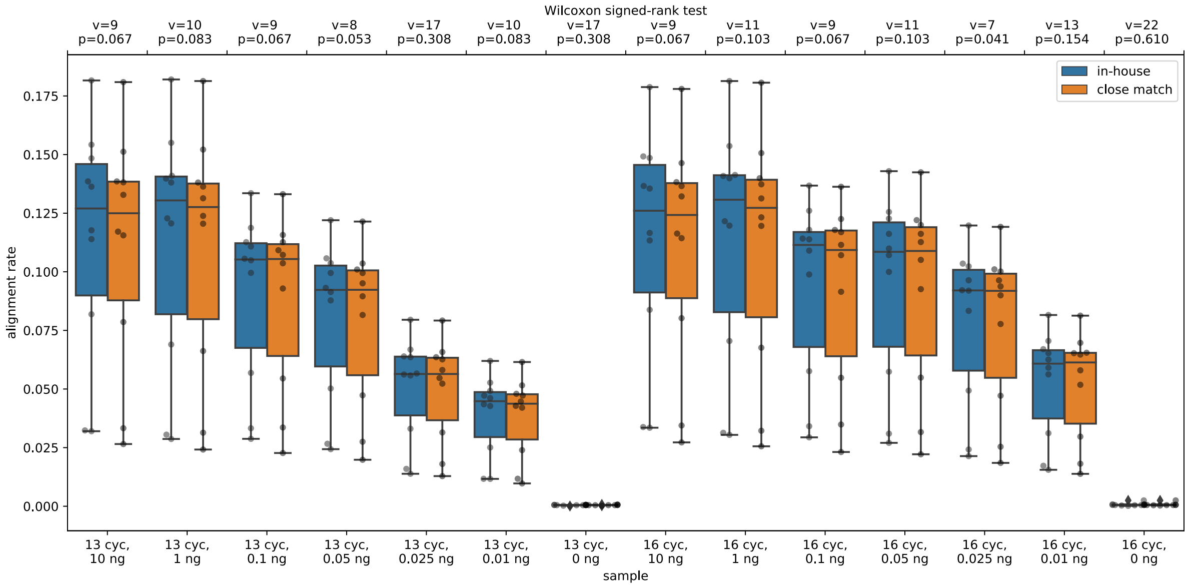

Supplementary Figure 7. Illumina sequencing for the pooled microbial reference community aligned to assembled genomes. Bwa mem alignment rates for microbial community data at different dilutions and PCR amplification cycles, generated using the Illumina iSeq100. Reads were aligned genomes for each of the ten strains in the reference community, using either in house assemblies or closely related strains. Alignment to in house assemblies tended to be slightly better, but not significantly so. Boxes span from the first to the third quartile, with medians denoted by a centre line, and whiskers represent the 1.5x interquartile range.

Supplementary References

1. Bankevich, A. *et al.* SPAdes: a new genome assembly algorithm and its applications to single-cell sequencing. *Journal of computational biology* **19**, 455–477 (2012).
2. Boetzer, M., Henkel, C. V., Jansen, H. J., Butler, D. & Pirovano, W. Scaffolding pre-assembled contigs using SSPACE. *Bioinformatics* **27**, 578–579 (2010).
3. Nadalin, F., Vezzi, F. & Policriti, A. GapFiller: a de novo assembly approach to fill the gap within paired reads. *BMC bioinformatics* **13**, S8 (2012).
