# Supplementary material for: Single-molecule sequencing detection of N6-methyladenine in microbial reference materials
Source: Nat Commun. 2019 Feb 4;10:579. doi: 10.1038/s41467-019-08289-9 (PMC6362088; doi:10.1038/s41467-019-08289-9)
Supplement: Supplementary file 2 — Reporting Summary [file 41467_2019_8289_MOESM2_ESM.pdf]

nature research

Corresponding author(s): Christopher E. Mason

Reporting Summary

Nature Research wishes to improve the reproducibility of the work that we publish. This form provides structure for consistency and transparency in reporting. For further information on Nature Research policies, see Authors & Referees and the Editorial Policy Checklist.

Statistical parameters

When statistical analyses are reported, confirm that the following items are present in the relevant location (e.g. figure legend, table legend, main text, or Methods section).

n/a	Confirmed	
☐	☒	The exact sample size (*n*) for each experimental group/condition, given as a discrete number and unit of measurement
☒	☐	An indication of whether measurements were taken from distinct samples or whether the same sample was measured repeatedly
☐	☒	The statistical test(s) used AND whether they are one- or two-sided<br>*Only common tests should be described solely by name; describe more complex techniques in the Methods section.*
☒	☐	A description of all covariates tested
☒	☐	A description of any assumptions or corrections, such as tests of normality and adjustment for multiple comparisons
☒	☐	A full description of the statistics including central tendency (e.g. means) or other basic estimates (e.g. regression coefficient) AND variation (e.g. standard deviation) or associated estimates of uncertainty (e.g. confidence intervals)
☒	☐	For null hypothesis testing, the test statistic (e.g. *F*, *t*, *r*) with confidence intervals, effect sizes, degrees of freedom and *P* value noted<br>*Give P values as exact values whenever suitable.*
☒	☐	For Bayesian analysis, information on the choice of priors and Markov chain Monte Carlo settings
☒	☐	For hierarchical and complex designs, identification of the appropriate level for tests and full reporting of outcomes
☒	☐	Estimates of effect sizes (e.g. Cohen's *d*, Pearson's *r*), indicating how they were calculated
☐	☒	Clearly defined error bars<br>*State explicitly what error bars represent (e.g. SD, SE, CI)*

Our web collection on statistics for biologists may be useful.

Software and code

Policy information about availability of computer code

Data collection	MinKNOW
Data analysis	albacore (v1.2.2 and v2.0.2), graphmap (v0.3.0), nanopolish (v0.8.4), mCaller (v0.3), Tombo (v1.2.1b), HOMER (v4.9.1), AME (v4.11.4), HGAP (v2), Canu (v1.5), SMRT Tools (v2.3.0 and v.5.0.1), BWA (v0.7.13 and v0.7.17), MACS2 (v2.1.1), Bismark (v0.7.12), PATRIC (v3.5), PHASTER (accessed February 2018) , pyani (v0.2.7), busco (v3.0.2), samtools (v1.7)

For manuscripts utilizing custom algorithms or software that are central to the research but not yet described in published literature, software must be made available to editors/reviewers upon request. We strongly encourage code deposition in a community repository (e.g. GitHub). See the Nature Research guidelines for submitting code & software for further information.

Data

Policy information about availability of data

All manuscripts must include a data availability statement. This statement should provide the following information, where applicable:

- Accession codes, unique identifiers, or web links for publicly available datasets
- A list of figures that have associated raw data
- A description of any restrictions on data availability

Sequence data (.fastq and .fast5 files) have been submitted to NCBI SRA with bioproject number PRJNA477598 [https://www.ncbi.nlm.nih.gov/bioproject/477598].

The Zymo microbial community strains are also included as regulatory-grade materials in the FDA-ARGOS repository at PRJNA231221 [https://www.ncbi.nlm.nih.gov/bioproject/231221] under sample names FDAARGOS_606 to FDAARGOS_612.

Field-specific reporting

Please select the best fit for your research. If you are not sure, read the appropriate sections before making your selection.

☒ Life sciences ☐ Behavioural & social sciences ☐ Ecological, evolutionary & environmental sciences

For a reference copy of the document with all sections, see nature.com/authors/policies/ReportingSummary-flat.pdf

Life sciences study design

All studies must disclose on these points even when the disclosure is negative.

Sample size	This was not a powered study.
Data exclusions	No data were excluded.
Replication	Model performance was assessed across a variety of species and with data from multiple labs.
Randomization	Samples/organisms/participants were not allocated into experimental groups.
Blinding	There was no group allocation.

Reporting for specific materials, systems and methods

Materials & experimental systems

n/a	Involved in the study	
☒	☐	Unique biological materials
☐	☒	Antibodies
☒	☐	Eukaryotic cell lines
☒	☐	Palaeontology
☒	☐	Animals and other organisms
☒	☐	Human research participants

Methods

n/a	Involved in the study	
☒	☐	ChIP-seq
☒	☐	Flow cytometry
☒	☐	MRI-based neuroimaging

Antibodies

Antibodies used	Synaptic Systems polyclonal rabbit anti-m6A (202 003)
Validation	The antibody has been validated for, from the product website, "human, rat, mouse, eukaryotes, prokaryotes" (https://www.sysy.com/products/m6a/facts-202003.php)
